# The dissociation between condom use knowledge and actual behavior among college students in Guangzhou, China: a cross-sectional study

**DOI:** 10.3389/frph.2026.1816452

**Published:** 2026-05-13

**Authors:** Yanjun Yang, Shaomin Wu, Yuan Tang

**Affiliations:** 1 Panyu District Center for Disease Prevention and Control, Guangzhou, China; 2 ISCTE Business School, BRU-IUL, Iscte University Institute of Lisbon of Lisbon, Lisbon, Portugal; 3 School of Health Management, Southern Medical University, Guangzhou, China; 4 Department of Medical Statistics, School of Public Health, Sun Yat-sen University, Guangzhou, China; 5 Department of Preventive Healthcare, Nansha District People’s Hospital, Guangzhou, China

**Keywords:** aids, behavior, college students, condom, knowledge

## Abstract

**Background:**

In the context of the progress made in HIV/AIDS prevention and treatment initiatives, the awareness of HIV/AIDS among Chinese adolescents has been on a steady upward trend. Nevertheless, the number of new HIV infections among college students keeps rising. In recent years, over 95% of newly diagnosed HIV/AIDS cases reported in China have been transmitted via sexual contact. In 2024, 100% of newly diagnosed HIV/AIDS cases in Guangzhou, China, have been sexually transmitted. One of the critical factors is the dissociation between condom use knowledge and actual behavior. That is to say, individuals have a comprehensive understanding of the transmission routes of AIDS, the infection modes, and the risks associated with unprotected sexual behavior, yet their actual sexual behavior goes against their awareness.

**Objectives:**

A comprehensive investigation was conducted to assess the current situation of the dissociation between condom use knowledge and actual behavior among college students. Moreover, relevant influencing factors were analyzed in great detail to lay a solid foundation for the exploration of highly effective intervention models.

**Methods:**

In November 2024, a cross-sectional survey was conducted at 13 universities and colleges in Guangzhou, China. Statistical analyses were performed using SPSS (version 26). A chi-square test and bivariate logistic regression analysis were utilized to compare the differences among respondents with diverse characteristics, and multivariate logistic regression was employed to analyze the primary influencing factors regarding the dissociation between condom use knowledge and actual behavior among college students.

**Results:**

Among the 1,583 sexually experienced respondents, 14.66% utilized condoms. Regarding demographic characteristics, when compared with females who were 22 years old or above, of Han ethnicity, residing in student dormitories, or having average monthly living expenses ranging from 1,000 to 2,000 RMB, males [odds ratio (OR) = 1.525, 95% confidence intervals (CI): 1.131–2.056], individuals aged 18–21 years (OR = 4.199, 95% CI: 1.352–13.045), ethnic minorities (OR = 1.981, 95% CI: 1.179–3.330), those living independently or sharing accommodation with their families (OR = 1.995, 95% CI: 1.286–3.093), or those with average monthly living expenses exceeding 3,000 RMB (OR = 3.470, 95% CI: 1.910–6.305) were more likely to show a dissociation between condom use knowledge and actual behavior among college students. Regarding sexual activity, alcohol consumption, and drug use, comparisons were made with non-homosexual individuals, those who had their first sexual intercourse at an age above 18 years, those who used a condom during their first sexual encounter with someone else, those who had sexual intercourse with only one partner, those with no experience of forced sexual intercourse, those with no casual sexual activities in the past year, those with no commercial sexual intercourse in the past year, or those whose frequency of alcohol consumption in the past three months was less than once a week. Homosexuality (OR = 2.149, 95% CI: 1.077–4.286), an age at first sexual intercourse of 18 or younger (OR = 1.557, 95% CI: 1.106–2.191), failure to use a condom during the first sexual intercourse (OR = 5.257, 95% CI: 3.592–7.693), having a total number of sexual partners ranging from 2 to 5 (OR = 2.356, 95% CI: 1.102–5.036), forced sexual intercourse (OR = 2.307, 95% CI: 1.199–4.436), casual sexual intercourse in the past year (OR = 2.407, 95% CI: 1.631–3.552), commercial sexual intercourse in the past year (OR = 17.885, 95% CI: 5.248–60.952), an alcohol consumption frequency in the past 3 months of at least once a week (OR = 2.140, 95% CI: 1.225–3.727) were more likely to exhibit a dissociation between condom use knowledge and actual behavior among college students. “The object of the first sexual intercourse not being a lover” was an independent protective factor against the dissociation between condom use knowledge and actual behavior (OR = 0.407, 95% CI: 0.197–0.842). When it came to HIV risk perception, related services, and attitudes, compared with statements such as “I don't know anyone with HIV”, “I haven't been tested for HIV”, “I'll get an HIV test if I engage in risky sexual behaviors”, and “I have a high level of HIV-related attitudes”, those who say “I know people with HIV (OR = 6.077, 95% CI: 3.220 –11.468)”, “I've been tested for HIV (OR = 2.840, 95% CI: 1.981–4.071)”, “People who wouldn't or were unsure if they'd get an HIV test after risky sexual behaviors (OR = 2.250, 95% CI: 1.634–3.099)”, and “Those with non-high HIV-related attitudes (OR = 1.698, 95% CI: 1.139–2.530)” are more likely to show a disconnect between condom use knowledge and behavior.

**Discussion and recommendations:**

This study revealed that nearly 15% of sexually active college students had experiences related to condom use and identified the factors influencing the dissociation between condom use knowledge and actual behavior. It is highly recommended to implement a multi-level intervention strategy targeting high-risk AIDS-related behaviors in order to decrease the incidence of high-risk sexual behaviors among college students.

## Introduction

Acquired Immunodeficiency Syndrome (AIDS) is a chronic infectious disease caused by the infection of the Human Immunodeficiency Virus (HIV). The HIV infects the human immune system, giving rise to immunodeficiency and ultimately resulting in death (1). AIDS has become one of the major global public health issues (2, 3). As of 2023, it was estimated that 39.9 million people were living with HIV globally (4). The World Health Organization (WHO) has set a target of limiting new HIV infections to 335,000 by 2030. However, the latest assessment indicates that attaining these objectives will not be straightforward (5). In 2023, approximately 1 million adolescents aged 15–19 years globally were living with HIV. Adolescents constitute around 3% of all individuals living with HIV and approximately 12% of adults newly infected with HIV. Outside of sub-Saharan Africa, Latin America and Asia have the highest number of adolescents with HIV (6). Adolescents account for an increasing proportion of the global HIV burden (7). As a sexually active population, young students are the key targets of HIV prevention and control in China (8, 9).

In recent years, around 3,000 young students in China have been reported infected with HIV each year. Sexual transmission is the main mode of transmission, and young students are facing significant challenges in epidemic prevention and control (10, 11). Previous studies have indicated that school health education courses can notably enhance students' knowledge of AIDS prevention and control (12). Moreover, there is a positive correlation between evidence-based health education and proactive health behaviors (13, 14). School-based health education programs targeting AIDS prevention have been widely promoted and implemented (15, 16). Moreover, students have demonstrated positive attitudes towards sexual health education (17, 18). It has been shown that individuals can adopt more preventive measures and reduce their risk of HIV infection by enhancing their knowledge in this domain (19, 20).

However, the effectiveness of preventing the spread of HIV merely through enhancing personal knowledge about HIV does not appear to be promising. According to several surveys conducted in China, approximately 25%–50% of the Chinese men who have sex with men (MSM) population exhibit a disconnection between knowledge and action regarding condom use (21–24). Regarding female sex workers (FSW), there exists a dissociation between their knowledge of HIV and condom use, presenting a phenomenon of a gap between knowledge and action (25, 26). Similarly, drug users also exhibit a separation between knowledge and action regarding AIDS (27). Among FSWs providing services in diverse settings across different regions, there exist varying rates of HIV awareness and condom use. Notably, the awareness rate of HIV knowledge is significantly higher than the rate of condom use during high-risk sexual behaviors. Specifically, 80.62%–99.31% of FSWs are aware of HIV/AIDS, while only 54.36%–81.47% of them used condoms during commercial sexual encounters with clients in the past month (28–31). A similar scenario prevails among other populations. Among Males who have casual sex with women (MCSW), 87.5%–98.22% are aware of HIV. However, in the past year, only 66.74%–71.40% consistently utilized condoms each time they had sexual intercourse with a commercial partner (32–34). The HIV prevalence rate among commercial sex clients in several Chinese cities ranged from 1.1% to 4.25% (33, 35–37). Specifically, in Guangdong Province, it was 4.25% (36). It has been found that different groups of individuals demonstrate their own unique characteristics in relation to the disconnection between knowledge and action. Specifically, as indicated in reference (37), the fact that people have received AIDS intervention services in the past year acts as a protective factor against the emergence of the knowledge-action disconnection among MSM. Additionally, young MCSWs who have received health education on sexually transmitted diseases are more likely to use condoms during sexual intercourse, as per reference (36). Nevertheless, the condom-use education aimed at drug users has not been able to significantly increase their condom-using rate, as reported in reference (38).

According to the monitoring data of the China Disease Control and Prevention Information System, as of December 31, 2024, there were more than 16,000 surviving HIV/AIDS cases in Guangzhou, China. From 2019 to 2024, among adolescents aged 15–24 in Guangzhou, the proportion of HIV/AIDS cases accounted for 3.5%–5.0% of the newly reported cases in that year, and HIV/AIDS cases among adolescents aged 15–24 constituted 19.3%–23.5% of the total cases. Meanwhile, there is a clear trend of the decreasing age of student cases. In 2024, the proportion of students aged 15–19 in the total number of student cases rose from 34.7% to 47.7%. Among the newly reported HIV infections in 2024, 51.7% of the infected individuals have a college degree or above. Furthermore, the highly educated MSM population in Guangzhou is a high-risk group for HIV infection and transmission. Previous survey findings have indicated that 41.1% of Chinese students reported using condoms (39). Moreover, the MSM student population shows insufficient awareness of their own vulnerability to HIV, along with a disconnection between knowledge and action (40). Additionally, the reported rate of MSM students who engaged in anal sex with men within the past year ranges from 38.99% to 58.42% (40).

The transition from forming cognitive attitudes to actual behavior change is a lengthy process. Prochaska et al. (41) proposed the Transtheoretical Model of Behavior Change, which categorizes behavioral transformation into five core stages: “precontemplation, contemplation, preparation, action, and maintenance.” While numerous scholars have conducted extensive research on HIV knowledge awareness, there is a dearth of studies examining the disconnect between prevention knowledge and behavior, with a notable research gap remaining among university students in Guangzhou, China. The disconnect between condom knowledge and actual behavior-where individuals understand HIV transmission risks but fail to consistently use condoms during high-risk sexual encounters-has become a critical bottleneck limiting the effectiveness of HIV education and behavioral interventions, and effectively improving the target population's HIV prevention behaviors to reduce infection risk has now become a vital component in interrupting HIV transmission within this group (42). China is characterized by a diverse demographic composition, consisting of 56 ethnic groups, along with rich cultural and linguistic diversity. In Guangzhou, China, certain universities admit students from various regions within China, encompassing the Chinese mainland, Hong Kong, Macau, and Taiwan, as well as those from the same general region but residing in different environments, such as urban, rural, and urban-rural areas. These students may exhibit variations in educational backgrounds and behaviors. Nevertheless, there has been a lack of adequate research regarding the dissociation between condom-use knowledge and behavior among college students in Guangzhou, China. Consequently, we conducted an investigation into the separation of condom use among college students in Guangzhou, China.

## Materials and methods

### Survey area

For this cross-sectional survey, we employed the Questionnaire Star online survey platform (https://www.wjx.cn) to collect data. The data was gathered from 13 universities in Guangzhou, China, in December 2024.

### Sample size

The sample size was calculated using the cross-sectional survey formula *n* = (z*_α_*^2^ × *pq*)/*d*^2^. Currently, there are few studies on the separation of condom use among college students. The sample size was calculated based on a previous survey indicating that the condom use rate among college students in Guangzhou ranges from 56.0% to 62.0% (43, 44). When *d* = 0.15*p*, *α* = 0.05, *z_α_* = 1.96, *q* = 1−*p*, *p* = 0.56, the sample size was 134 individuals; when *p* = 0.62, it was 104 individuals.

### Study design, participants, and procedures

We will use the method of stratified cluster random sampling to select 13 universities in Guangzhou, China, including 3 comprehensive universities, 3 medical universities, 3 polytechnic and technical science and technology universities, 2 art universities, 1 normal university and 1 language university. Then, we will randomly select all students in each grade from the undergraduate students of the selected schools and no less than 2 classes of graduate students from each school. In December 2024, the survey respondents will fill in the Internet electronic questionnaire through the QR code on their mobile phones, and each mobile phone number can only fill in one questionnaire. The first paragraph of the questionnaire outlines the purpose of the study, procedures, anonymity and strict confidentiality. To enhance participant engagement, this survey was conducted collaboratively by the university's student affairs department and health services division.

## Measures

### Socio-demographic measures

Through the survey, socio-demographic statistics are gathered, encompassing the following aspects: gender, age, ethnicity (including the Han ethnicity and ethnic minorities), type of educational institution (categorized as comprehensive universities, medical universities, polytechnic and technical universities, art universities, normal universities, and language universities), academic major (classified as science, management, medicine, education, engineering, literature, or other majors), academic level (divided into undergraduate levels, master's degrees, or doctorates), accommodation type (classified as student dormitories, living with family, shared accommodation, or living alone), family residence location (categorized as cities, townships, urban-rural areas, or rural areas), family structure (classified as intact family, restructured family, divorced family, or others), place of origin (divided into the Chinese mainland, Hong Kong, Macau, or Taiwan), and average monthly living expenses (classified as less than 1,000 RMB, 1,000–3,000 RMB, or more than 3,000 RMB).

### Measures of sexual behavior, alcohol consumption, and drug use

Statistical data on the respondents' sexual behavior and alcohol consumption were collected. This includes aspects like sexual orientation, the age of first sexual intercourse, types of sexual partners (such as spouse, heterosexual partners, or others), the age gap between the respondents and their sexual partners (either within 10 years or over 10 years), whether the first sexual activity was with a lover (yes or no), whether a condom was used during the first sexual encounter with someone else (yes or no), the total number of sexual partners (categorized as 1, 2–5, or 6 or more), experience of forced sexual activity (yes or no), experience of sexual masturbation (yes or no), previous online browsing of sexual or sexually explicit content (yes or no), use of online channels like social media, websites, or online games to find sexual partners (yes or no), occurrence of temporary sexual activity in the past year (yes or no), occurrence of commercial sexual activity in the past year (yes or no), the nature of relationships with sexual partners (classified as spouses, cohabiting heterosexual partners, temporary sexual partners, commercial sexual partners, or same-sex sexual partners), frequency of condom use during sexual activity (classified as every time, sometimes, or never), frequency of alcohol consumption in the past three months (categorized as less than once a week or once a week or more), and drug use experience (yes or no).

### Measures of HIV risk perception and related services

The survey gathered data regarding the respondents' HIV risk perception, related services, and attitudes. This included inquiries such as whether they perceived themselves to be at risk of HIV infection (yes/no), whether they knew individuals living with HIV (yes/no), whether they had undergone HIV testing (yes/no), whether they would seek HIV testing if engaged in sexual activities (yes/no), whether they had participated in HIV prevention and control education (yes/no), and whether they had taken part in related activities such as HIV prevention and STD prevention programs offered by schools (yes/no).

### Measures of HIV-related knowledge and attitudes

**Figure 1:**
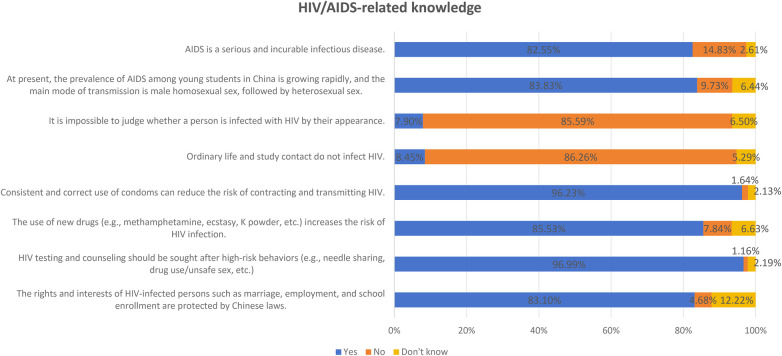
HIV/ADIS-related knowledge.

In this study, we used the “AIDS Knowledge Questionnaire for Young Students” (45) developed by the AIDS Prevention and Control Center of the Chinese Center for Disease Control and Prevention (STD) to assess the participants' knowledge of HIV/AIDS. The questionnaire consists of 8 questions, each with three response options: yes, no, or not sure (for example, “Consistent and correct use of condoms can reduce the risk of contracting and transmitting HIV”) (see Figure 1). To evaluate the respondents' attitudes toward HIV/AIDS, we utilized the Adolescent HIV/AIDS-related Attitudes Questionnaire developed by Huang et al. (46). The questionnaire contains a total of 24 questions, including both positive and negative ones, and employs a 5-point Likert scale. For example, for positive questions, “strongly agree” is scored as 5, “agree” as 4, “uncertain” as 3, “disagree” as 2, and “strongly disagree” as 1. For negative questions, the scoring is reversed. The higher the score, the more positive the attitude. Attitudinal levels are also determined using a three-level scale: a high-level score ranges from 89 to 120, a medium-level score ranges from 57 to 88, and a low-level score ranges from 24 to 56. The CVI (Index of Content Validity) of each item in the questionnaire was between 0.80 and 1.00, the average CVI of all items was 0.96, and the Cronbach's alpha coefficient of the questionnaire was 0.79.

### Statistical methods

Respondents who were capable of correctly answering the question “Can consistent and correct use of condoms reduce the risk of contracting and transmitting HIV?” were included in the study on the separation of knowledge and action regarding condom use. For data analysis, SPSS (version 26) was employed. Percentages and 95% confidence intervals were utilized to characterize categorical variables. The chi-square test was used to compare the disparities between categorical variables. The OR with 95% CI was calculated by designating “condom use knowledge and action separation (Yes = 1, No = 0)” as the dependent variable, and factors with a *P* < 0.1 in the bivariate logistic regression analysis were integrated into the multivariate logistic regression model. The significance level was set at 0.05 (two-tailed).

### Ethical approval and participant consent

The research protocol was meticulously reviewed and approved by the Academic Committee of the Panyu District Center for Disease Control and Prevention, Panyu District, Guangzhou City, Guangdong Province, China [Reference Number: Panyu CDC Yilun 2023-001]. The research procedures strictly complied with human research guidelines, including the Declaration of Helsinki. All participants signed an online informed consent form. Data collection was carried out anonymously and analyzed using a numeric code.

## Results

### Demographic characteristics of cognitively correct condom use

A total of 1,645 college students reported engaging in sexual behavior. This accounted for 13.02% of the total surveyed population (1,645/12,632, 95% CI: 12.44%–13.61%). The average score for HIV/AIDS-related knowledge was 7.00 (SD = 1.38) out of a maximum of 8 points, with an overall correct percentage of 89.69%. Among these students, 1,583 participants (96.23%, 95% CI: 95.31%–97.15%) answered correctly regarding condom usage awareness. There were 812 male students (51.30%) and 771 female students (48.70%).905 individuals (57.17%) were aged between 19 and 22 years. There were 982 undergraduates, accounting for 62.03% of the total number of students. Among the 601 graduate students (37.97%), 79.70% were master's students. 90.02% of the students lived mainly in student dormitories, and 79.15% of them had an average monthly living allowance ranging from 1,000 to 3,000 RMB (see Table 1, Figure 1).

**Table 1:** General demographic characteristics of college students with correct cognition of condom use.

Item	Category	*n*	%
Sex	Male	812	51.30
	Female	771	48.70
Age (years)	<18	17	1.07
	18-	145	9.16
	19-	207	13.08
	20-	246	15.54
	21-	230	14.53
	22-	222	14.02
	≥23	516	32.60
Nationality	Han	1,486	93.87
	Minority	97	6.13
School type	Comprehensive University	246	15.54
	Medical University	412	26.03
	Polytechnic and Technological University	511	32.28
	Art University	90	5.69
	Normal university	65	4.11
	Language University	259	16.36
Specialty	Engineering	469	29.63
	Medicine	352	22.24
	Science	180	11.37
	Literature	154	9.73
	Management	120	7.58
	Pedagogy	106	6.70
	Others	202	12.76
Current stage of study	Freshman	236	14.91
	Sophomore	236	14.91
	Junior	266	16.80
	Senior	244	15.41
	Postgraduate	479	30.26
	Doctoral	122	7.71
Lodging	Student dormitories	1,425	90.02
	Living with family/Shared flats/Living alone	158	9.98
Registered permanent residence	City	1,105	69.80
	Township/Urban-rural junction	283	17.88
	Countryside	195	12.32
Family status	Family of origin	1,388	87.68
	Combined families	74	4.67
	Divorced families	105	6.63
	Others	16	1.01
Region of source of students	Chinese mainland	1,504	95.01
	Hong Kong/Macau/Taiwan	79	4.99
Average monthly living expenses (yuan)	<1,000	65	4.11
	1,000–2,000	1,253	79.15
	>3,000	265	16.74
Frequency of alcohol consumption in the 3 months (per week)	<1	1,482	93.62
	≥1	101	6.38
Drug abuse experience	Yes	7	0.44
	No	1,576	99.56

### HIV/AIDS-related attitudes

**Figure 2:**
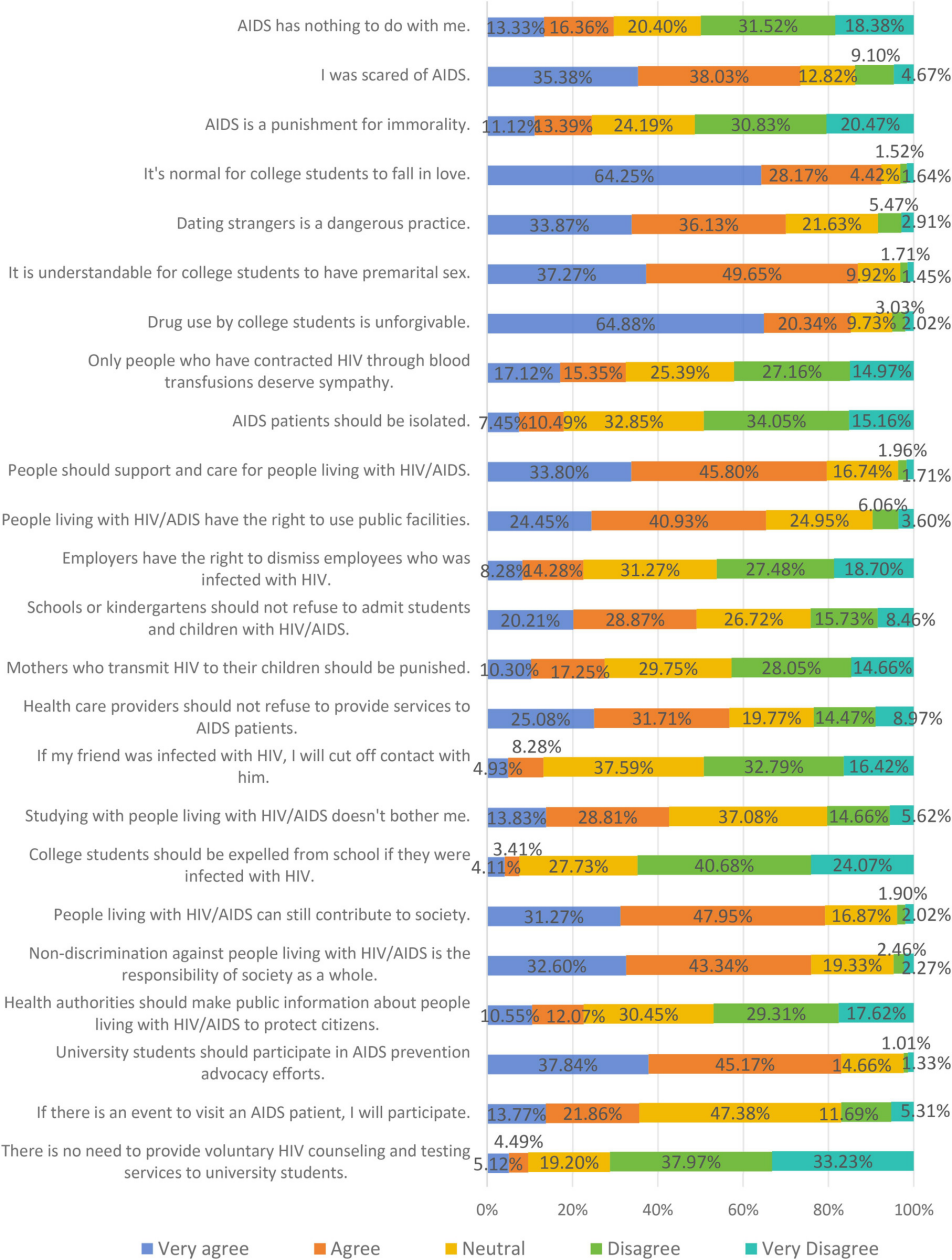
HIV/AIDS-related attitude.

Among the 1,583 respondents with knowledge of condom use, the mean score for HIV-related attitudes was 58.92 (SD = 9.69) out of a maximum of 120 points. The low, medium, and high levels of overall attitude constituted 0.63%, 77.70%, and 21.67% respectively. Moreover, 73.40% of the respondents reported being “afraid of AIDS”, 24.51% believed that “AIDS is a punishment for immoral behavior”, 74.42% considered that “dating strangers is a dangerous practice”, 17.94% held the opinion that “AIDS patients should be isolated”, and 7.52% believed that “college students should be expelled from school if infected with AIDS” (see Figure 2).

#### Factors influencing the dissociation between knowledge of condom use and actual behavior

Among the 1,583 respondents with knowledge of condom use, 232 (14.66%, 95% CI: 12.91%–16.40%) did not show consistency in condom use, while 1,351 (85.34%, 95% CI: 83.60%–87.09%) did. Among those who did not show consistency (condom separatists), 35 were MSM, accounting for 49.30% (35/71, 95% CI: 37.38%–61.21%) of the total number of MSM in this group.

When it comes to demographic characteristics, variables like gender, age, ethnicity, accommodation type, and average monthly living expenses were significantly correlated with the gap between condom use knowledge and actual behavior. Compared with females, people aged 22 and above, of Han ethnicity, those “living in student dormitories” and those “living in student dormitories with an average monthly living expenditure of 1,000–2,000 RMB”, males (OR = 1.525, 95% CI: 1.131–2.056), individuals aged between 18 and 21 years (OR = 4.199, 95% CI: 1.352–13.045), ethnic minorities (OR = 1.981, 95% CI: 1.179–3.330), those “living with family, living alone or sharing a house” (OR = 1.995, 95% CI: 1.286–3.093) and those with “average monthly living expenses over 3,000 yuan” (OR = 3.470, 95% CI: 1.910–6.305) were more likely to show a gap in condom use (see Table 2).

**Table 2:** Logistic regression analysis of demographic characteristics and the separation of knowledge and action of condom use in college students.

Item	Category	Bivariable regression analysis				Multivariable regression analysis			
		*B*	*P*	OR	95% CI	*B*	*P*	OR	95% CI
Sex	Female (Ref.)								
	Male	0.434	0.003	1.544	1.161–2.054	0.422	0.006	1.525	1.131–2.056
Age (years)	≥22 (Ref.)								
	18–21	1.669	0.001	5.307	1.969–14.307	1.435	0.013	4.199	1.352–13.045
	<18	1.244	0.013	3.470	1.298–9.272	1.002	0.067	2.725	0.933–7.960
Nationality	Han (Ref.)								
	Minority	0.578	0.023	1.782	1.084–2.930	0.684	0.010	1.981	1.179–3.330
School type	Normal university (Ref.)								
	Polytechnic and Technological University	0.748	0.082	2.114	0.909–4.913	0.194	0.676	1.214	0.489–3.016
	Art University	0.670	0.001	1.954	1.294–2.952	0.623	0.078	1.864	0.933–3.725
	Medical University	0.413	0.224	1.511	0.777–2.937	−0.133	0.729	0.876	0.412–1.859
	Language University	0.384	0.068	1.468	0.972–2.218	0.247	0.402	1.280	0.719–2.279
	Comprehensive University	0.276	0.233	1.318	0.838–2.074	−0.055	0.862	0.947	0.512–1.752
Specialty	Science (Ref.)								
	Management	0.953	0.002	2.593	1.420–4.737	0.437	0.240	1.548	0.747–3.208
	Medicine	0.890	0.011	2.434	1.227–4.828	0.682	0.079	1.978	0.925–4.230
	Pedagogy	0.538	0.020	1.713	1.088–2.697	−0.272	0.476	0.762	0.361–1.607
	Engineering	0.348	0.271	1.416	0.763–2.628	0.279	0.451	1.322	0.640–2.729
	Literature	0.433	0.044	1.542	1.011–2.351	0.207	0.462	1.230	0.708–2.138
	Others	0.333	0.231	1.395	0.809–2.406	0.502	0.124	1.652	0.871–3.133
Current stage of study	Postgraduate (Ref.)								
	Freshman	0.325	0.275	1.384	0.773–2.480	0.360	0.369	1.433	0.653–3.145
	Sophomore	0.026	0.909	1.027	0.655–1.610	0.086	0.733	1.089	0.667–1.780
	Junior	0.762	0.001	2.143	1.394–3.293	0.396	0.230	1.486	0.778–2.835
	Senior	0.624	0.012	1.867	1.148–3.038	0.513	0.053	1.670	0.993–2.809
	Doctoral	0.422	0.084	1.525	0.945–2.459	0.191	0.505	1.210	0.691–2.120
Lodging	Student dormitories (Ref.)								
	Living with family/Shared flats/Living alone	0.778	<0.001	2.177	1.475–3.214	0.691	0.002	1.995	1.286–3.093
Region of source of students	Chinese mainland (Ref.)								
	Hong Kong/Macau/Taiwan	0.576	0.038	1.779	1.031–3.068	0.058	0.875	1.060	0.513–2.189
Average monthly living expenses (yuan)	1,000–3,000 (Ref.)								
	>3,000	1.149	<0.001	3.156	1.800–5.536	1.244	<0.001	3.470	1.910–6.305
	<1,000	0.179	0.559	1.197	0.656–2.183	0.455	0.166	1.576	0.828–2.997
Registered permanent residence	Countryside (Ref.)								
	Township/Urban-rural junction	0.314	0.185	1.369	0.860–2.179				
	City	0.166	0.391	1.180	0.808–1.724				
Family status	Family of origin (Ref.)								
	Others (Combined families, Divorced families)	0.196	0.340	1.217	0.813–1.822				

With reference to sexual behavior, alcohol consumption, and drug use, factors including sexual orientation, “the age of first sexual intercourse”, “the partner of first sexual intercourse”, “whether condoms were utilized during the first sexual encounter with another individual”, “the number of sexual partners in previous relationships”, “whether one has been coerced into sexual intercourse”, “whether commercial sexual intercourse has occurred in the past year”, “the frequency of alcohol consumption in the past three months (per week)”, and “drug use experience” are significantly correlated with the dissociation between condom use knowledge and actual behavior. In comparison with individuals who were “non-homosexual,” had an “age at first sexual intercourse > 18 years old,” used condoms during their first sexual intercourse, had sexual relations with only one person, had no experience of forced sexual intercourse, had no casual sexual encounters in the past year, had no commercial sexual activities in the past year, and had an alcohol consumption frequency of less than once a week in the past three months, men who have sex with men (MSM) with an odds ratio (OR) of 2.149 (95% CI: 1.077–4.286), those with an age at first sexual intercourse ≤18 years (OR = 1.557, 95% CI: 1.106–2.191), those who did not use condoms during their first sexual intercourse (OR = 5.257, 95% CI: 3.592–7.693), those with a total number of sexual partners ranging from 2 to 5 (OR = 2.356, 95% CI: 1.102–5.036), those who had experienced forced sexual intercourse (OR = 2.307, 95% CI: 1.199–4.436), those who had casual sexual intercourse in the past year (OR = 2.407, 95% CI: 1.631–3.552), those who had commercial sexual intercourse in the past year (OR = 17.885, 95% CI: 5.248–60.952), and those with an alcohol frequency of at least once a week in the past three months (OR = 2.140, 95% CI: 1.225–3.727) were more likely to demonstrate a gap in condom use. “The object of the first sexual intercourse not being a lover” was an independent protective factor against the discrepancy between condom use knowledge and actual behavior (OR = 0.407, 95% CI: 0.197–0.842) (see Table 3).

**Table 3:** Logistic regression analysis of sexual behaviour, alcohol consumption, and condom use among university students.

Item	Category	Bivariable regression analysis				Multivariable regression analysis			
		B	P	OR	95% CI	B	P	OR	95% CI
Sexual orientation	Others (Ref.)								
	Homosexual	1.870	<0.001	6.490	3.981–10.580	0.765	0.030	2.149	1.077–4.286
Age at the time of first sexual activity (Year)	>18 (Ref.)								
	≤18	1.028	<0.001	2.796	2.104–3.716	0.443	0.011	1.557	1.106–2.191
The object of the first sexual intercourse	Spouse/Cohabiting heterosexualromantic parner (Ref.)								
	Others	1.452	<0.001	4.273	2.782–6.565	−0.308	0.473	0.735	0.316–1.705
The age of the person who had sex for the first time (Year)	The age difference is within 10 years (Ref.)								
	The age difference is more than 10 years	1.887	<0.001	6.600	3.349–13.007	0.330	0.554	1.392	0.465–4.161
Use the internet or social media to find someone for the first sexual intercourse	No (Ref.)								
	Yes	1.252	<0.001	3.499	2.477–4.942	0.141	0.600	1.152	0.679–1.954
The sexual intercourse is with a lover	Yes								
	No	1.063	<0.001	2.895	1.989–4.213	−0.899	0.015	0.407	0.197–0.842
Condoms were used during the first sexual intercourse	Yes (Ref.)								
	No	1.696	<0.001	5.452	3.977–7.474	1.659	<0.001	5.257	3.592–7.693
Total number of sexual partners	1 (Ref.)								
	2–5	2.563	<0.001	12.971	7.779–21.628	0.857	0.027	2.356	1.102–5.036
	≥6	1.747	<0.001	5.736	3.356–9.803	0.482	0.197	1.619	0.778–3.367
Whether have been forced to have sex	No (Ref.)								
	Yes	1.842	<0.001	6.312	4.062–9.809	0.836	0.012	2.307	1.199–4.436
Sexual masturbation	Never/Occasional (once every few months or longer) (Ref.)								
	Frequent (once every few weeks or less)	1.083	<0.001	2.953	2.016–4.326	0.112	0.670	1.118	0.669–1.868
	Every day or several times a week	0.677	0.002	1.968	1.288–3.007	−0.089	0.740	0.915	0.543–1.543
Have ever browsed sexual or pornographic information on the web	No (Ref.)								
	Yes	0.699	<0.001	2.012	1.403–2.884	0.184	0.394	1.202	0.874–2.842
Ever used the Internet to find a sexual partner (social media/websites/online games, etc.)	No (Ref.)								
	Yes	1.870	<0.001	6.489	4.502–9.352	0.455	0.130	1.576	0.874–2.842
Have had temporary sexual activity in the past year	No (Ref.)								
	Yes	1.414	<0.001	4.112	3.046–5.551	0.878	<0.001	2.407	1.631–3.552
Have had commercial sex in the past year	No (Ref.)								
	Yes	3.987	<0.001	53.880	18.855–153.968	2.884	<0.001	17.885	5.248–60.952
Frequency of alcohol consumption in the last 3 months (per week)	<1次 (Ref.)								
	≥1次	1.089	<0.001	2.973	1.905–4.638	0.761	0.007	2.140	1.228–3.727
Drug abuse experience	No (Ref.)								
	Yes	2.698	0.001	14.857	2.865–77.038	0.667	0.569	1.949	0.197–19.326

Regarding HIV risk perception, associated services, and attitudes, “the presence of individuals with HIV within one's social circle,” “previous experience of HIV testing,” “willingness to undergo HIV testing following risky sexual behaviors,” and “attitudes related to AIDS” exhibit a significant correlation with the dissociation between condom use knowledge and actual behavior. Compared with those who didn't know anyone with HIV, those who knew people with HIV (OR = 6.077, 95% CI: 3.220–11.468), those who had undergone HIV testing (OR = 2.840, 95% CI: 1.981–4.071), those who would get tested for HIV if they had risky sexual behavior (OR = 2.840, 95% CI: 1.981–4.071) as opposed to those who wouldn't or were unsure, and those with non-high levels of HIV-related attitudes (OR = 1.698, 95% CI: 1.139–2.530) were more likely to have a dissociation between condom use knowledge and actual behavior (see Table 4).

**Table 4:** Logistic analysis of AIDS risk perception, related services, attitudes and the separation of condom knowledge and action.

Item	Category	Bivariable regression analysis				Multivariable regression analysis			
		*B*	*P*	OR	95% CI	*B*	*P*	OR	95% CI
Believe I am at risk of HIV infection	No (Ref.)								
	Yes	0.424	0.005	1.528	1.137–2.055	0.115	0.485	1.122	0.812–1.551
Among the people I know, there are people living with AIDS	No (Ref.)								
	Yes	2.162	<0.001	8.693	4.844–15.598	1.805	<0.001	6.077	3.220–11.468
Have been tested for HIV	No (Ref.)								
	Yes	1.057	<0.001	2.876	2.063–4.010	1.044	<0.001	2.840	1.981–4.071
If you're having risky sex, will you get tested for HIV?	Yes (Ref.)								
	No/Uncertain	0.653	<0.001	1.921	1.416–2.605	0.811	<0.001	2.250	1.634–3.099
HIV/AIDS-related attitudes	High level (Ref.)								
	Non-high level	0.546	0.005	1.727	1.175–2.539	0.517	0.009	1.698	1.139–2.530
Ever received education on HIV/AIDS prevention and control in school	Yes (Ref.)								
	No	0.294	0.146	1.341	0.903–1.993				
Participated in the school's AIDS prevention and STD prevention activities	Yes (Ref.)								
	No	0.287	0.122	1.333	0.926–1.918				

### Frequency of condom use

Respondents reported how frequently they used condoms with various sexual partners. The total proportion of respondents who used condoms every time they had sex was 72.50%, which was statistically significantly different from the frequency of condom use during sexual intercourse with different sexual partners (*χ*^2^ = 66.691, *P* < 0.001). Compared with other sexual partners, the proportion of spouses or cohabiting heterosexual partners was the highest (77.50%), and the proportion of commercial sexual partners was the lowest (20%), and the difference was statistically significant (*P* < 0.001) (Table 5).

**Table 5:** Comparison of the frequency of condom use during sex with different subjects in the last year.

Sexual partner	Every time *n* (%)	Sometimes or never *n* (%)	*χ* ^2^	*P*
Spouse, cohabiting heterosexualromantic parner^a^^,^^b^^,^^c^ (*n* = 809)	627 (77.50)	182 (22.50)	66.691	<0.001
Temporary sexual partners^a^^,^^d^ (*n* = 208)	136 (65.38)	72 (34.62)		
Commercial sexual partners^b^^,^^d^^,^^e^ (*n* = 30)	6 (20.00)	24 (80.00)		
Gay sexual partners^c^^,^^e^ (*n* = 51)	27 (52.94)	24 (47.06)		
Total	796 (72.50)	302 (27.50)		

a,b,c,d Bonferroni pairwise comparison results, the same letter indicates that the difference is statistically significant (*P* < 0.001).

e Bonferroni pairwise comparison results, the same letter indicates that the difference is statistically significant (*P* < 0.01).

The proportion of respondents experiencing condom separation was relatively high during sexual intercourse with their spouses or co-habiting heterosexual partners (*χ*^2^ = 21.445, *P* < 0.001), temporary sexual partners (*χ*^2^ = 6.668, *P* < 0.01), and gay male sexual partners (*χ*^2^ = 6.246, *P* < 0.01) (Table 6).

**Table 6:** Frequency of condom use compared to the separation between knowledge and action of AIDS during sexual activity in the past year.

Sexual partner	The separation between knowledge and action of AIDS	Every time *n* (%)	Sometimes or never *n* (%)	*χ* ^2^	*P*
Spouse,cohabiting heterosexualromantic parner^a,b,c^ (*n* = 809)	Yes	77 (61.60)	48 (38.40)	21.445	<0.001
	No	550 (80.41)	134 (19.89)		
	Total	627 (77.50)	182 (22.50)		
Temporary sexual partners^a,d^ (*n* = 208)	Yes	18 (47.37)	20 (52.63)	6.668	0.010
	No	118 (69.41)	52 (30.59)		
	Total	136 (65.38)	72 (34.62)		
Commercial sexual partners^b,d,^^e^ (*n* = 30)	Yes	2 (33.33)	4 (66.67)	0.883	0.361
	No	4 (16.67)	20 (83.33)		
	Total	6 (20.00)	24 (80.00)		
Same-sex sexual partners^c,^^e^ (*n* = 51)	Yes	3 (23.08)	10 (76.92)	6.246	0.012
	No	24 (63.16)	14 (36.84)		
	Total	27(52.94)	24(47.06)		

^a,b,c,d^ Bonferroni pairwise comparison results, the same letter indicates that the difference is statistically significant (*P* <0.001).

^e^ Bonferroni pairwise comparison results, the same letter indicates that the difference is statistically significant (*P* <0.01).

## Discussion and recommendations

The findings of this study indicate that among the college students surveyed who reported having had sexual experiences, over 70% said they were afraid of AIDS and considered dating strangers a risky behavior. The proportion of those with a gap between their knowledge and practice of condom use was 14.66%, which is lower than the 41.1% reported in a 2019 survey by Tan et al. (39) of 1,303 undergraduates from 6 universities in Zhuhai City, China, and also lower than the 35% reported in a survey by Tan et al. (47) from 2019 to 2021 of 759 undergraduates from 21 universities in Chongqing City, China. In this study, 49.30% of MSM reported a dissociation between condom use knowledge and actual behavior, which is higher than the 40.8% found in a 2017–2018 survey by Gao et al. (48) of 549 university-student MSM in Harbin, Tianjin, Xi'an, and Chongqing. Previous research has demonstrated that having knowledge of AIDS acts as a protective factor against HIV infection (49), while inconsistent condom use poses a risk of contracting HIV (50). A two-year study conducted by Chen et al. (51) in China, targeting college freshmen for an intervention on AIDS-related knowledge and behaviors, found that without intervention, there was no significant change in college students' AIDS knowledge levels. In the meantime, the number of students with sexual experience gradually increased, leading to a gap between knowledge and behavior. These results suggest that when formulating AIDS prevention and control policies, policymakers should not only concentrate on enhancing the knowledge of the targeted group but also take additional steps to decrease the incidence of risky behaviors among them.

This study reveals that among the surveyed college students, those who claimed to have found sexual partners online faced a relatively high risk of a disparity between condom-related knowledge and actual behavior. The utilization of dating apps simplifies the process for college students to initiate sexual contact (52), and those who search for sexual partners on social media are more prone to engage in high-risk sexual activities (53). Some scholars have proposed that online platforms for seeking sexual partners can serve as effective channels for knowledge dissemination (54). Currently, new media is developing at a rapid pace. Policymakers should make better use of online promotion platforms to intervene in risky behaviors among specific active populations. Among the respondents in this study, drug users are more likely to have a gap in condom knowledge, which is consistent with the findings of Thepthien et al. (55) who reported that cannabis use is significantly associated with risky sexual behaviors (RSB). A survey by Shi et al. (56) among college students in 56 universities in China shows that the reasons for drug use among college students are influenced by various factors, including personal ones (curiosity, vanity, profit-seeking, etc.) and objective ones (insufficient anti-drug education in universities, difficulty in drug identification, negative impacts of drug subculture and family environment, etc.). How to combine AIDS prevention and control with anti-drug efforts on campus warrants further discussion in future research.

The findings of the multi-factor logistic regression analysis indicate that there's a fairly wide gap between condom knowledge and behavior among 18–21-year-old males and MSM. Previous studies have indicated that MSM tend to use condoms less consistently during anal sex, which makes them a high-risk group for HIV infection (57). Moreover, the disconnection between their condom-related knowledge and actual behavior is quite common (58, 59). Young men who have sex with men (YMSM), a significant subgroup within MSM, experience a more pronounced disconnection between knowledge and action (48, 60); leading to a higher prevalence of HIV infection (61, 62). The incidence rate of HIV among MSM aged 18–24 is 2.5 times that of older age groups (63). The research findings of Envuladu et al. (64) conducted on 428 Nigerian adolescents aged 18–19 indicate that approximately one-third of these adolescents are sexually active, and the majority of them do not use condoms consistently during sexual intercourse. Male adolescents are more prone than female adolescents to use condoms consistently during sexual behavior (65–67). There are differences in sexual sub-cultures among gay and bisexual men (GBM) within the MSM population (68). The receptive partner role (often referred to as the “female role”) is the most prevalent (69). Among MSM, those in the insertive partner role (the “male role”) have a higher proportion of bisexuality and tend to be more dominant in condom-use decisions, which makes unprotected sex more likely (70). Interventions targeting MSM populations should also consider the characteristics associated with bisexuality (71). Enhancing the self-protective awareness of insertive MSM is especially crucial for increasing condom usage rates and reducing the risk of sexually transmitted diseases (72). These results suggest that in the future, when formulating intervention policies for risky behaviors among young students, the government should fully consider the characteristics of different gender-identity groups. The aim is to improve the risk perception of those who have the decision-making power on condom use and prompt them to take appropriate protective measures.

This study demonstrates that among the surveyed college students, individuals who “reside with their families, live independently, or share housing” display a substantially broader disparity between condom-related knowledge and actual behavior when compared to those residing in campus dormitories. However, certain scholars have reported contradictory results. For example, Mihret Melese et al. (73) found in their research on Ethiopian students aged 11–18 that living with family serves as a protective factor against adolescents' involvement in high-risk sexual activities, and those not living with family are nearly twice as likely to engage in such high-risk behavior. Two studies on Ethiopian adolescents aged 14–19 showed that parental monitoring serves as a protective factor against high-risk sexual behaviors (74). This might be because family supervision deters adolescents from participating in such high-risk activities (75). This study indicates that individuals with a monthly living expenditure exceeding 3,000 yuan are more prone to encounter a disparity between knowledge and action. This finding aligns with the report by Tan et al., which suggests that a monthly expenditure of 3,000 yuan or more serves as a risk factor for the disconnection between knowledge and action in relation to AIDS. This seems to suggest that high-income groups are highly susceptible to the knowledge-action gap. A rise in living costs implies more social activities, which, in turn, further elevates the likelihood of engaging in risky behaviors.

The results of the multifactor logistic regression analysis also indicate that individuals who “had their first sexual experience before the age of 18”, “didn't use a condom during their first sexual encounter with a partner”, “had casual sex in the past year”, and “participated in commercial sexual activities in the past year” are more prone to having a condom-use disconnect. Previous research has demonstrated that younger partners often struggle to convince older sexual partners to use condoms, particularly when the partner is reluctant to do so (50, 76, 77). “Using condoms during the first sexual encounter” is closely linked to adolescents' subsequent sexual behaviors as part of early sexual experiences (78). A study by Nascimento et al. (79) on 193 medical students in Brazil showed that not using condoms with casual partners in the past 12 months was related to the respondents' poor knowledge of sexually transmitted diseases. However, for most respondents with low knowledge levels, knowledge wasn't the only factor determining risky behaviors. The condom-use rate in casual relationships is significantly lower than that in stable relationships (80, 81). In this study, respondents who had previously accessed sexual or pornographic content online were at a relatively higher risk of a gap between their knowledge of condom use and its actual practice. This is in line with the findings of Mukanga et al. (82), which showed that 12th-grade students in Zambia who watched porn movies were more likely to engage in high-risk sexual behavior. Among the respondents in this study, those with “two or more sexual partners” faced a relatively higher risk of a difference between their knowledge and the actual use of condoms. This is consistent with the results of some studies indicating a correlation between condom use and the number and type of sexual partners (83). People with multiple or non-monogamous partners were more likely to have unprotected sex (84–86). Meanwhile, this study shows that people with “2–5 sexual partners” have a risk of a gap between condom knowledge and actual use that's almost 13 times that of those with “just one sexual partner”. However, when the number of sexual partners goes over 6, the risk of this knowledge-use gap seems to go down. This seems to suggest there's a tipping point; maybe when the number of sexual partners reaches a certain level (like 6 or more), people may switch from being driven by sexual impulses to caring more about the risks associated with sexual activity and taking proper safety measures. This calls for further research and discussion.

The findings of this study indicate that people who've had at least one drink a week over the past three months and gone through forced sexual activity are more likely to have a disparity between what they know about condom use and their actual behavior. Previous research has demonstrated that alcohol consumption decreases drinkers' inclination to use condoms (87). A study by Papas et al. (88) on 507 sexually-active, HIV-infected drinkers in western Kenya found that both the occurrence and frequency of drinking are linked to unprotected sex. Alcohol use is a risk factor for adolescents engaging in high-risk sexual behavior (89). Young students are more likely to engage in high-risk sexual activities after drinking (84, 85), and this tendency is more evident in males (89, 90). This result suggests that when implementing interventions for risky behaviors among college students, public education can incorporate the harms of excessive drinking to the body. Previous research has shown that intimate partner violence (IPV) and sexual violence (SV) are major public health concerns for women, especially female college students. Two studies from universities in the northeastern United States found that 36%–52% of female college students have experienced IPV/SV, and 8%–22% of the respondents reported experiencing IPV/SV in the past six months (91, 92). IPV can make victims fear their sexual partners, reducing their willingness to advocate for condom use and thus increasing the risk of unintended pregnancies and sexually transmitted infections among female college students (93). This further suggests that efforts to prevent AIDS should be integrated with the promotion of legal regulations. At the same time, the All-China Women's Federation, a mass organization dedicated to safeguarding the legitimate rights and interests of women and children, should demand and assist relevant departments or units in investigating actions that violate the rights and interests of women and children, offer help to the affected women and children, and better play its role in advocating for the legitimate rights of female university students.

The findings of this study suggest that individuals who know someone with HIV, have previously been tested for HIV, or are uncertain about getting tested after engaging in risky behavior, as well as those with a low level of positive attitudes towards HIV-related matters, are more likely to experience a disparity between their knowledge and condom-use behavior. Generally, college students often have unstable sexual relationships. Even if one partner is regarded as stable, there's no guarantee that this partner doesn't have other sexual partners. Thus, the risk of contracting HIV through unprotected sex still exists (94, 95). Choi et al. (96) found that interventions aimed at preventing HIV-related behaviors among 18–24-year-old MSM who seek partners online show that behavior hinges on individual attitudes and beliefs. Additionally, there is no significant correlation between how often participants engage in knowledge education and their behavioral changes. It is recommended that relevant organizations, when providing knowledge education and evaluating college students' risky behaviors, should also focus on cultivating positive attitudes among college students.

Our study has several limitations that should be acknowledged. First, our sample was restricted to university students in Guangzhou, China, so the findings should be interpreted with caution when generalizing to student populations in other geographical regions. Second, participant recall bias may have compromised the accuracy of the data. Third, respondents might have misreported sexual behavior due to privacy concerns, although anonymous electronic data collection was implemented to mitigate reporting bias to some extent. For future research, expanding the geographical scope would provide a more comprehensive understanding of regional variations across China, thereby offering empirical support for developing targeted high-risk behavior interventions. Additionally, exploring effective strategies to promote behavioral change regarding high-risk sexual activities among university students represents a valuable area for further investigation.

In summary, the study findings indicate that there's a disparity between college students' knowledge of condoms and their actual condom-using behavior, regardless of their level of safe-sex knowledge. For college students with relatively high AIDS knowledge, merely amassing knowledge isn't sufficient to alter their attitudes and beliefs towards risky behaviors. Promoting and encouraging condom use is a vital component of interventions for AIDS-related high-risk behaviors. Relevant government departments and decision-makers should formulate targeted behavioral intervention measures based on the characteristics of the college student population and the local real-life learning environments. Meanwhile, they should enhance AIDS education to improve students' knowledge levels, aiming to decrease the incidence of risky behaviors and consequently reduce the transmission of sexually transmitted diseases and AIDS.

## Data Availability

The original contributions presented in the study are included in the article/Supplementary Material, further inquiries can be directed to the corresponding author.

## References

[1] Weinberg JL Kovarik CL. The WHO clinical staging system for HIV/AIDS. AMA J Ethics. (2010) 12(3):202–6. 10.1001/virtualmentor.2010.12.3.cprl1-1003 23140869

[2] Money DM. HIV/AIDS is not over. J Obstet Gynaecol. (2022) 44(12):1240–1. 10.1016/j.jogc.2022.10.007 36567089

[3] Thornton J. Botswana’s HIV/AIDS success. Lancet. (2022) 400(10351):480–1. 10.1016/S0140-6736(22)01523-9 35964598

[4] UNAIDS. Latest global and regional HIV statistics—fact sheet 2024 (2024).

[5] WHO. World Health Statistics 2024: Monitoring Health for the SDGs, Sustainable Development Goals. Geneva: WHO (2024).

[6] UNICEF. Adolescent HIV prevention: In order to ramp up our efforts in the fight against AIDS, there is a need for more concentrated focus on adolescents and young people (2024).

[7] HIV/AIDS JUNPo. The Path That Ends AIDS: UNAIDS Global AIDS Update 2023. Geneva, Switzerland: UNAIDS (2023).

[8] He N. Research progress in the epidemiology of HIV/AIDS in China. China CDC Weekly. (2021) 3(48):1022–30. 10.46234/ccdcw2021.249 34888119 PMC8633551

[9] Zhang P Zou J Gao CZ. Research progress on epidemic characteristics of AIDS among young students in China. Occup Health. (2022) 38(16):2295–8. 10.13329/j.cnki.zyyjk.2022.0494

[10] Han MJ Chen QF Xu P Shi Y. Strive for a new journey in AIDS prevention and control during the 13th five-year: review and prospect of AID Sprevention and control in China. Chin J AIDS STD. (2021) 27(12):1327–31. 10.13419/j.cnki.aids.2021.12.01

[11] Cai C Tang HL Chen FF Li DM Lyu P. Characteristies and trends of newly reported HIV infecting in young students in China, 2010–2019. Chin J Epidemiol. (2020) 41(9):1455–9. 10.3760/cma.j.cn112338-20200417-00592 33076598

[12] Liu Y Lu L Wang YY Wilkinson MR Ren YM Wang CC Effects health education HIV/AIDS related knowledge among first year university students in China. Afi Health Sci. (2020) 20(4):1582–90. 10.4314/abs.v20i4.10 PMC8351845 34394218

[13] Kalichman SC Rompa D. Functional health literacy is associated with health status and health-related knowledge in people living with HIV-AIDS. J AIDS. (2000) 25(4):337–44. 10.1016/s0749-3797(00)00121-5 11114834

[14] Duh E Medina SP Coppersmith N Adjei N Roberts MB Magee S. Sex ed by brown med: a student-run curriculum and its impact on sexual health knowledge. Fam Med. (2017) 49(10):785–8. 29190404

[15] Oppong AK Oti-Boadi M. HIV/AIDS knowledge among undergraduate university students: implications for health education programs in Ghana. Afr Health Sci. (2013) 13(2):270–7. 10.4314/ahs.v13i2.11 24235924 PMC3824504

[16] Kim HY Park M Lee E. A cross-sectional survey of relationships between sexual knowledge, sexual attitudes, and reproductive health behaviour among female university students. Contemp Nurse. (2018) 54(6):640–50. 10.1080/10376178.2018.1556104 30513057

[17] Ayalew M Nigatu D Sitotaw G Debie A. Knowledge and attitude towards sexual and reproductive health rights and associated factors among Adet Tana Haik College students, Northwest Ethiopia: a cross-sectional study. BMC Res Notes. (2019) 12(1):80. 10.1186/s13104-019-4116-4 30755247 PMC6373003

[18] Halstead V Williams JR Gonzalez-Guarda R. College Students’ perspectives on campus health centers as a sexual assault resource: a qualitative analysis. Violence Vict. (2018) 33(1):109–25. 10.1891/0886-6708.33.1.109 29195514

[19] Oyeyemi YA Abdulkarim A Oyeyemi BO. The influence of knowledge and sociodemographics on AIDS perception and sexual practices among secondary school students in Nigeria. Afr Health Sci. (2011) 11(1):S67–76.20. 10.4314/ahs.v11i3.70073 22135648 PMC3220127

[20] Siuki HA Peyman N Vahedian-Shahroodi M Gholian-Aval M Tehrani H. Health education intervention on HIV/AIDS prevention behaviors among health volunteers in healthcare centers: an applying the theory of planned behavior. J Soc Serv Res. (2019) 45(4):582–8. 10.1080/01488376.2018.1481177

[21] Dong WB Zhao JX Li SF Wang XW Chen LY Cai Y Results and the factors of separation between knowledge and action among cross-sectional survey of men who had sex with men in Yuxi Prefecture, Yunnan Province, 2010–2019. Chin J Dermatovenereol. (2021) 35(2):160–6. 10.13735/j.cjdv.1001-7089.202009004

[22] Lu YZ Nong QX Nong LP Factors associated with the inconsistency between cognition and sexual behaviors among MSM. Chin J Dis Control Prev. (2015) 19(12):1231–1234, 1239. 10.16462/j.cnki.zhjbkz.2015.12.012

[23] Wang Y Li LL Zhang GG The separation conditions of beliefs from behaviors among men who have sex with men: an approach for influencing factors. Chin J Dis Control Prev. (2014) 18(10):951–5.

[24] Huang XD Zhang HL Wang R Awareness of AIDS knowledge and behavior characteristics among men who have sex with men in Xi'an city, 2016–2019. South China J Prev Med. (2021) 47(2):146–9.

[25] Wu H Zhou Y Xu YQ. Influencing factors of condom usage among older female sex workers in Qingdao city. Chin J Public Health. (2017) 33(3):352–6.

[26] Yang QW Ding JQ Liu JM. A KAP study on AIDS among 814 FSWs in Ningbo City, 2011–2019. Chin J Health Educ. (2021) 37(11):999–1004. 10.16168/j.cnki.issn.1002-9982.2021.11.009

[27] Li LY Chen XM Liu YJ Huang LH. Sentinel surveillance results of HIV/AIDS among drug abusers in Dali Prefecture from 2016 to 2022. Jiangsu J Prev Med. (2024) 35(5):661–3. 10.13668/j.issn.1006-9070.2024.05.033

[28] Wang ZX Wei S Zhang HQ. Analysis of AIDS, syphilis knowledge, behaviors and infection related factors among female sex workers. Chin J Public Health Manag. (2016) 32(6):794–796, 823. 10.19568/j.cnki.23-1318.2016.06.007

[29] He B Guo D Nong LP Analysis on factors influencing AIDS infection and condom use in female sex workers aged ≥50 years old in Nanning City from 2017–2019. Occup Health. (2022) 38(2):202–9. 10.13329/j.cnki.zyyjk.2022.0078

[30] Zeng W Yang YJ Liang S Awareness of AIDS knowledge and high-risk behavior characteristics of female sex workers in Panyu District, Guangzhou, 2017–2019. South China J Prev Med. (2021) 47(3):335–8.

[31] Shi Y Zhang XY Wang GX Analysis of HIV/AIDS sentinel surveillance results among female sex workers in Fangshan District of Beijing from 2016 to 2020. Occup Health Damage. (2021) 36(6):368–72.

[32] Wang YH Zhou YH Cen P Analysis on AIDS related knowledge, high-risk behaviors and STDs infection status of whoremasters in Kaiyuan City, Yunnan Province. Chin J Dis Control Prev. (2019) 23(3):268–72. 10.16462/j.cnki.zhjbkz.2019.03.005

[33] Yang D Jiang FS Xu F Analysis of the results of HIV/STD surveillance among prostitutes in Guilin from 2015 to 2019. South China J Prev Med. (2022) 48(1):74–76, 80.

[34] Li Y Liao B Li J An analysis of the sentinel surveillance of AIDS among commercial sex clients in Kunming city from 2010–2015. J Kunming Med Univ. (2019) 40(2):139–44.

[35] Zhu J Hu D Yin Y Zhu Z Wang N Wang B. HIV Prevalence and correlated factors among male clients of female sex worker in a border region of China. PLoS One. (2019) 14(11):e0225072. 10.1371/journal.pone.0225072 31697754 PMC6837524

[36] Zhao PZ Shen HC Huang SJ Analysis on the status and related factors of high risk behaviors of sexually transmitted diseases/HIV among clients of female sex workers in Guangdong Province. Chin J Dis Control Prev. (2018) 22(1):100–3. 10.16462/j.cnki.zhjbkz.2018.01.023

[37] Yu T Yang YZ Wang JL Sentinel surveillance of AIDS among 400 commercial sex clients in Liuzhou City in 2015. Chin J Dis Control Prev. (2016) 20(12):1261–4. 10.16462/j.cnki.zhjbkz.2016.12.018

[38] Zeng LM Ai Y Zhuo LP. HIV/AIDS-related knowledge, behavior, and infection rate of drug abusers in Jingmen City. J Public Health nd Prev Med. (2013) 24(5):80–1.

[39] Tan XX Zhou Y Feng SX Dai WC Huang ZQ Liu YW Analysis of associated factors of the inconsistency for knowledge and behavior in condom use among college students in Zhuhai City. Chin J School Health. (2023) 44(10):1497–1500, 1504. 10.16835/j.cnki.1000-9817.2023.10.013

[40] Cai YH Wei HF Wang ZF Zhang TX Zhang MM. AIDS Knowledge awareness and HIV detection rates among college students who have sex with men. Chin J Sch Doctor. (2024) 38(10):721–724, 732. 10.20161/j.cnki.32-1199/R.202410200

[41] Prochaska JO Velicer WF. The transtheoretial model of health behavior change. Am J Health Promot:12:38–48. 10.4278/0890-1171-12.1.38 10170434

[42] Wan SP Wang GY Zheng SF. Causes and responses for separation of HIV/AIDS knowledge and behaviors. Pract J Clin Med. (2014) 11(3):123–5.

[43] Luo JB Yang YJ. Change trends in AIDS-related knowledge and sexual behaviors among senior university students in Guangzhou city. Chin J Public Health. (2019) 35(5):598–602. 10.11847/zgggws1122778

[44] Chen YY Yang YJ Lang S Xiong TJ Meng WY He QX. Survey on the current situation of AIDS knowledge, attitude and behavior of first-grade students of an art university in Guangzhou. J Med Pest Control. (2021) 37(5):446–9. 10.7629/yxdwfz202105010

[45] National Center for AIDS/STD Control and Prevention, China CDC. Questionnaire on HIV/AIDS awareness among young students. Available online at: https://www. chinaaids.cn/qsnazbfk/xzzx/201705/P020170510598644914191.pdf (Accessed May 12, 2025).

[46] Huang J. HIV/AIDS Knowledge, Attitudes, and High-risk behavior Education and Effect Evaluation of College Students. Beijing, China: Chinese Academy of Medical Sciences, Peking Union Medical College (2007).

[47] Tan B Bu QQ Chen XR Zhang M Deng D. Separation situation of AIDS prevention knowledge and behavior among school youth with self-reported sexual behavior in Chongqing Municipality. Chin J AIDS&STD. (2023) 29(11):1220–4. 10.13419/j.cnki.aids.2023.11.11

[48] Gao D Wu J Zhang WJ Chen TQ Cui WX Hu YF HIV Knowledge and high-risk sexual behaviors of men who have sex with men in college students men in college students. Chin J Sch Health. (2019) 40(3):359–63. 10.16835/j.cnki.1000-9817.2019.03.012

[49] Chen WL Chen JH Zhang H Behavioral characteristics and factors relate to HIV infection among Gay/Bisexual Men in Fuzhou. Chin J AIDS&STD. (2022) 28(8):910–4. 10.13419/j.cnki.aids.2022.08.07

[50] Morris L Kouya F Kwalar R Pilapil M Saito K Palmer N Factors associated with inconsistent condom use in adolescents with negative or unknown HIV status in Northwest Cameroon. AIDS Care. (2014) 26(11):1440–5. 10.1080/09540121.2014.920948 24865769 PMC4122626

[51] Chen LX Xiang XC Fei Y Wan YT Qin JJ Guo JQ Research on the necessity of AIDS intervention for college students based on cognitive behavioral therapy. Curr HIV Res. (2022) 20(6):430–40. 10.2174/1570162X20666220822111050 35996265

[52] Xu J Luo Y Dong H Zhao G. The effects of internet exposure on sexual risk behavior among sexually experienced male college students in China: cross-sectional study. JMIR Public Heal Surveill. (2022) 8(5):e3184. 10.2196/31847 PMC9112083 35499864

[53] Deogan C Jacobsson E Mannheimer L Björkenstam C. Meeting sexual partners online and associations with sexual risk behaviors in the Swedish population. J Sex Med. (2020) 17(11):2141–7. 10.1016/j.jsxm.2020.08.001 32873533

[54] Hollingshead BM Dowsett GW Bourne A. ‘It’s like getting an Uber for sex’: social networking apps as spaces of risk and opportunity in the Philippines among men who have sex with men. Health Sociol Rev. (2020) 29:264–78. 10.1080/14461242.2020.1820366 33411604

[55] Thepthien BO Celyn. Risky sexual behavior and associated factors among sexually-experienced adolescents in Bangkok, Thailand: findings from a school web-based survey. Reprod Health. (2022) 19(1):127. 10.1186/s12978-022-01429-3 35643503 PMC9148491

[56] Shi JH Li JJ. The investigation into the causes of drugs-relatedness among university students and its countermeasures. J Liaoning Univ (Phil Soc Sci). (2022) 50(6):78–86. 10.16197/j.cnki.lnupse.2022.06.013

[57] Mustanski B Ryan DT Newcomb ME D’Aquila RT Matson M. Very high HIV incidence and associated risk factors in a longitudinal cohort study of diverse adolescent and young adult men who have sex with men and transgender women. AIDS Behav. (2020) 24(6):1966–75. 10.1007/s10461-019-02766-4 31858300 PMC8560086

[58] Coelho LE Torres TS Veloso VG Grinsztejn B Jalil EM Wilson EC The prevalence of HIV among men who have sex with men (MSM) and young MSM in Latin America and the Caribbean:a systematic review. AIDS Behav. (2021) 25(10):3223–37. 10.1007/S10461-021-03180-5 33587242

[59] Bai JY Ning TL Zhou N Guo Y Yi MH. HIV Infection status and related factors in men who have sex with men in sentinel surveillance in Tianjin. Chin J Epidemiol. (2019) 40(9):1106–10. 10.3760/cma.j.issn.0254-6450.2019.09.016 31594154

[60] Cao BL Zhao PP Bien C Pan S Tang WM Watson J Linking young men who have sex with men (YMSM) to STI physicians:a nationwide cross-sectional survey in China. BMC Infect Dis. (2018) 18(1):288. 10.1186/s12879-018-3145-2 29776395 PMC5960109

[61] Schackman BR. The value and challenge of frequent human immunodeficiency virus (HIV) testing among young men who have sex with men in the United States. Clin Infect Dis. (2021) 73(7):e1936–7. 10.1093/cid/ciaa1060 32730624 PMC8492148

[62] Fan S Yang ZY Hou FS Yu MH Luo ZZ Liao MZ HIV and syphilis and sexual risk behaviours among men who have sex with men attending university in China:a systematic review and meta-analysis. Sex Health. (2019) 16(6):554–65. 10.1071/SH18231 31570116

[63] Sullivan PS Rosenberg ES Sanchez TH Explaining racial disparities in HIV incidence in black and white men who have sex with men in Atlanta, GA:a prospective observational cohort study. Ann Epidemiol. (2015) 25(6):445–54. 10.1016/j.annepidem.2015.03.006 25911980 PMC4433604

[64] Esther EA Massar K Wit JBF. Diversities of sexual activities and correlates of safe sex practices among adolescents in Plateau State, Nigeria. Front Reprod Health. (2021) 3:744622. 10.3389/frph.2021.744622 36303998 PMC9580655

[65] Katikiri E Njau B. Motivating factors and psychosocial barriers to condom use among out-of-school youths in Dar es Salaam, Tanzania: a cross sectional survey using the health belief model. ISRN AIDS. (2012) 2012:170739. 10.5402/2012/170739 24052872 PMC3767346

[66] Bhana D Anderson B. Gender, relationship dynamics and South African girl’s vulnerability to sexual risk. Afr J AIDS Res. (2013) 12:25–31. 10.2989/16085906.2013.815408 25871308

[67] Darteh EKM Dickson KS Doku DT. Women’s reproductive health decision-making: a multi-country analysis of demographic and health surveys in sub-Saharan Africa. PLoS One. (2019) 14:e0209985. 10.1371/journal.pone.0209985 30625212 PMC6326492

[68] Schnarrs PW Jones SS Parsons JT Baldwin A Rosenberger JG Lunn MR Sexual subcultures and HIV prevention methods: an assessment of condom use, PrEP, and TasP among gay, bisexual, and other men who have sex with men using a social and sexual networking smartphone application. Arch Sex Behav. (2021) 50(4):1781–92. 10.1007/s10508-020-01784-x 32728870 PMC10388693

[69] Lin CZ Zhang H Chen JH He DS Xue HH Xu SY. Analysis of behavioral characteristics among the men who have sex with men with different sexual roles. Chin J AIDS & STD. (2020) 30(10):1052–6. 10.13419/j.cnki.aids.2024.10.09

[70] Chen JH Zhang H Xue SG Xue HH. Characteristics and HIV infection related factors of men who have sex with men and bisexual sexual orientation in Fuzhou from 2016–2021. Mod Dis Control Prev. (2024) 35(3):179–83. 10.13515/j.cnki.hnjpm.1006-8414.2024.03.007

[71] Larmarange J Broqua C. Les hommes bisexuels sont moins exposés au virus de l’immunodéficience humaine que les homosexuels exclusifs en Afrique subsaharienne. Sante Publique. (2023) 34(HS2):123–32. 10.3917/spub.hs2.0123 37336726

[72] Robbins SJ Dauda W Kokogho A Ndembi N Mitchell A Adebajo S Oral sex practices among men who have sex with men and transgender women at risk for and living with HIV in Nigeria. PLoS One. (2020) 15(9):e0238745. 10.1371/journal.pone.0238745 32886722 PMC7473579

[73] Melese M Esubalew D Siyoum TM Worku YB Azanaw J Mengistie BA. Parent-adolescent communication on sexual and reproductive health issues and associated factors among secondary public-school students in Gondar town, northwest Ethiopia: an institution based cross-sectional study. Front Public Health. (2024) 19(12):1342027. 10.3389/fpubh.2024.1342027 PMC11407295 39290406

[74] Wondimagegne YA Anbese AT. Risky sexual behaviors and associated factors among adolescent in Gedeo Zone, South Ethiopia: a community based cross-sectional study. Sci Rep. (2024) 14(1):19908. 10.1038/s41598-024-67944-4 39198585 PMC11358130

[75] Dadi AF Teklu FG. Risky sexual behavior and associated factors among grade 9–12 students in Humera secondary school, western zone of Tigray, NW Ethiopia. Sci J Public Health. (2014) 5(2):410–6. 10.11648/j.sjph.20140205.16

[76] Amo-Adjei J. Age differences and protected first heterosexual intercourse in Ghana. Afr J Reprod Health. (2012) 16(4):58–67. 23444544

[77] Dietrich J Sikkema K Otwombe KN Sanchez A Nkala B Bruyn G Multiple levels of influence in predicting sexual activity and condom use among adolescents in Soweto, Johannesburg, South Africa. J HIV AIDS Soc Serv. (2013) 12(3-4):404–23. 10.1080/15381501.2013.819312 24532992 PMC3920549

[78] Oidtman J Sherman SG Morgan A German D Arrington-Sanders R. Satisfaction and condomless anal sex at sexual debut and sexual risk among young black same-sex attracted men. Arch Sex Behav. (2017) 46(4):947–59. 10.1007/s10508-016-0831-2 27649695 PMC5581662

[79] Nascimento MCS Junior LRCF Freitas ICF Avena KM Andrade BB. Relationship between sexually transmitted infections knowledge and the sexual behavior of Brazilian future doctors. Front Med (Lausanne). (2024) 11:1512590. 10.3389/FMED.2024.1512590 39776844 PMC11703906

[80] Gomes NL Lopes CS. Panorama of risky sexual behaviors in the Brazilian adult population-PNS 2019. Rev Saude Publica. (2022) 56:61. 10.11606/s1518-8787.2022056004007 35766790 PMC9239426

[81] Wildsmith E Manlove J Steward-Streng N. Relationship characteristics and contraceptive use among dating and cohabiting young adult couples. Perspect Sex Reprod Health. (2015) 47:27–36. 10.1363/47e2515 25581462

[82] Mukanga B Dlamini SB Mwanabute N Taylor M. Adolescents’ risky sexual behaviours and practices: implications for sexuality education implementation in Zambia. Afr J Prim Health Care Fam Med. (2024) 16(1):e1–11. 10.4102/phcfm.v16i1.4476 39099271 PMC11304187

[83] Chen Q Sun YM Sun WD Hao MQ Li GY Su XL Trends of HIV incidence and prevalence among men who have sex with men in Beijing, China:nine consecutive cross-sectional surveys, 2008–2016. PLoS One. (2018) 13(8):e0195982. 10.1371/journal.pone.0201953 30092072 PMC6084969

[84] Srahbzu M Tirfeneh E. Risky sexual behavior and associated factors among adolescents aged 15–19 years at governmental high schools in aksum town, tigray, Ethiopia, 2019: an institution-based, cross-sectional study. Biomed Res Int. (2020) 2020:3719845. 10.1155/2020/3719845 32904524 PMC7456495

[85] Ojukwu EN Okoye HU Saewyc E. Social correlates of HIV-risky behaviours among African Canadian adolescents living in British Columbia, Canada:a secondary data analysis. Int J Environ Res Public Health. (2023) 20(11):6031. 10.3390/ijerph20116031 37297635 PMC10252849

[86] Ren Z Zhou Y Liu Y. Factors associated with unsafe sexual behavior among sexually active Chinese University students, Hebei Province, 2019. BMC Public Health. (2021) 21(1):1904. 10.1186/s12889-021-11992-2 34670556 PMC8529721

[87] Wolfs K Bos AER Mevissen FEF Lankveld JJDM. The effect of alcohol and sexual arousal on explicit and implicit condom attitudes and intentions to use a condom. Arch Sex Behav. (2023) 52(4):1715–25. 10.1007/S10508-022-02511-4 36441371 PMC10125951

[88] Papas RK Gakinya BN Mwaniki MM Wu XK Lee H Martino S Associations with unprotected sexual behavior among HIV-infected drinkers in Western Kenya. AIDS Behav. (2018) 22(9):2840–50. 10.1007/s10461-018-2150-1 29767325 PMC6239984

[89] Cho HS Yang Y. Relationship between alcohol consumption and risky sexual behaviors among adolescents and young adults: a meta-analysis. Int J Public Health. (2023) 68:1–21. 10.3389/ijph.2023.1605669 PMC10154531 37153699

[90] Strandberg A Skoglund C Gripenberg J Kvillemo P. Alcohol and illicit drug consumption and the association with risky sexual behaviour among Swedish youths visiting youth health clinics. NAD Nord Stud Alcohol Drugs. (2019) 36:442–59. 10.1177/1455072519845970 PMC7434140 32934578

[91] Sutherland MA Fantasia HC Hutchinson MK. Screening for intimate partner and sexual violence in college women: missed opportunities. Women’s Health Issues. (2016) 26(2):217–44. 10.1016/j.whi.2015.07.008 26329257

[92] Sutherland MA Hutchinson MK. Intimate partner and sexual violence screening practices of college health care providers. Appl Nurs Res. (2018) 39:217–9. 10.1016/j.apnr.2017.11.031 29422162 PMC5810590

[93] Peasant C Sullivan TP Ritchwood TD Parra GR Weiss NH Meyer JP Words can hurt: the effects of physical and psychological partner violence on condom negotiation and condom use among young women. Women Health. (2018) 58(5):483–97. 10.1080/03630242.2017.1316345 28402194 PMC5638679

[94] Liu Y Russ S Mitchell J Przybyla S Zhang C. Assessing the determinants of quality of life and the impact on HIV prevention measures among HIV-negative and status-unknown young men who have sex with men:a study in two U.S. metropolitan areas. Int J Environ Res Public Health. (2022) 19(2):726. 10.3390/IJERPH19020726 35055548 PMC8776199

[95] Janulis P Goodreau SM Birkett M Phillips G Morris M Mustanski B Temporal variation in one-time partnership rates among young men who have sex with men and transgender women. J Acquir Immune Defic Syndr. (2021) 87(3):e214–21. 10.1097/QAI.0000000000002679 33675616 PMC8192435

[96] Choi SK Golinkoff J Michna M Connochie D Bauermeister J. Correlates of engagement within an online HIV prevention intervention for single young men who have sex with men: randomized controlled trial. JMIR Public Health Surveill. (2022) 8(6):e33867. 10.2196/33867 35759333 PMC9274398

